# Translation and validation of the Arabic version of the Morisky, Green and Levine (MGL) adherence scale

**DOI:** 10.1371/journal.pone.0275778

**Published:** 2022-10-07

**Authors:** Oriana Awwad, Suha AlMuhaissen, Ayat Al-Nashwan, Salahdein AbuRuz

**Affiliations:** 1 Department of Biopharmaceutics and Clinical Pharmacy, School of Pharmacy, University of Jordan, Amman, Jordan; 2 Department of Pharmaceutics and Pharmaceutical Technology, School of Pharmacy, University of Jordan, Amman, Jordan; 3 Department of Pharmacology and Therapeutics, College of Medicine and Health Sciences, United Arab Emirates University, Al Ain, United Arab Emirates; PLOS: Public Library of Science, UNITED KINGDOM

## Abstract

The Morisky Green Levine (MGL) adherence scale is a 4-item tool used for the detection of medication nonadherence among patients with chronic health conditions. Despite being widely used in Arabic-speaking research contexts, it has never been validated in Arabic language. The aim of this study was to translate and validate the MGL tool into Arabic. A standard forward-backward process was used to translate the questionnaire. Cronbach’s alpha coefficient was measured to assess internal consistency of the scale. The test-retest reliability measured the consistency of participants’ responses over time. Construct validity was evaluated by Explanatory factor analysis (EFA); Kaiser-Meyer-Olkin value and Bartlett’s test of sphericity were determined. Convergent validity was assessed using a preexisting medications Arabic Adherence Assessment Tool (AAAT). The model fit was evaluated using confirmatory factor analysis (CFA). Associations between the MGL scale scores and the patient demographic/clinical characteristics were tested by linear regressions. A total of 201 participants were included into the study. The MGL scale categorization revealed that 20.9%, 59.2% and 19.9% of the participants had high, moderate and low levels of adherence respectively. Adequate internal consistency (alpha = 0.593) was observed. A significant strong ICC and Pearson’s correlations were generated between responses at time 1 and time 2. EFA results elucidated the suitability of the data for factor analysis. Pearson’s coefficient (r) revealed a significant strong correlation between MGL scale and AAAT. CFA results confirmed a good fit for the suggested model. Linear regression revealed higher number of medications, more frequent outpatient clinic visits and not experiencing medication adverse effect factors significantly associated with better adherence. The Arabic version of MLG scale is a reliable valid tool to assess adherence among Arabic-speaking communities. Implementing interventions targeting patients not compliant to regular clinic visits and those at higher risk of experiencing medication side effects can greatly enhance medication adherence.

## Introduction

With the increased prevalence of chronic diseases requiring long-term treatment, and the concern of medication nonadherence growing to healthcare systems, there is crucial need for valid and reliable tools for the easy assessment of patients’ adherence in a busy clinical practice setting [1, 2].

Available data revealed that adherence to medications among patients suffering from chronic diseases averages 50% in the developed countries [3]. In the MENA region, including Jordan, these rates have been showed to be even lower [4–10]. In a study conducted among Jordanians receiving long-term treatment for different conditions, most of the participants showed to have poor adherence to their treatments [4]. When looking to patients taking chronic medications for specific chronic diseases such as COPD, asthma or osteoporosis, the results were comparable, also demonstrating low rates of adherence [5–7]. In the MENA region, few studies reporting results from different middle eastern countries confirmed the presence of nonadherence as a problem among patients with chronic diseases [8–10]. Poor adherence to medications results in failure of treatment, in 33–69% of medicine-related hospital admissions, and in approximately $100 billion health care-related costs annually, necessitating screening of patients for nonadherence [3].

Direct and objective measures of adherence such as measurement of drug concentration in the body fluids, the use of databases and the Medication Events Monitoring System (MEMS) are considered accurate methods but retain drawbacks in being expensive, difficult to perform and not reveling any pattern or cause of nonadherence [1]. On the other hand, subjective measures such as the use of questionnaires and scales, despite being less accurate, are the most popular approaches and most commonly used in clinical practice due to their low cost, simplicity, rapid administration and real-time feedback [1].

The Morisky, Green and Levine (MGL) scale is a 4-item adherence tool initially developed and validated in patients with hypertension [11]. Later, the scale has been used to assess adherence to medication among patients with different chronic conditions [1, 12]. The tool is considered the quickest and easiest to administer and the "yes-saying" bias approach allows disclosures of patients’ nonadherence [1, 11, 12]. In addition, it has been validated in the broadest range of chronic conditions including patients with low health literacy making it the adherence tool most widely used in research [1, 12].

While the other adherence scales developed by Prof. Morisky are copyrighted, the MGL scale is in the public domain and has been largely cited in the last decade in several research studies, including studies from the MENA region and Jordan [5–7, 13–29].

Despite the wide use of the MGL scale in Arabic-speaking research contexts, this tool has never been validated in Arabic language. Not a unique valid Arabic version of the scale exists, which limits its utilization as a reliable tool. This study thus aims to translate and validate the 4-item Morisky Green Levine adherence scale into Arabic language to have a unified validated version to be used among Arabic-speaking communities.

## Methods

### Study design and participants

This validation study was conducted at a tertiary governmental hospital in Amman. Amman is the biggest city and the capital of Jordan with the largest population [30]. A convenience sampling method was employed at the outpatient clinics of the site hospital. This allowed a variety of patients with different clinical and medication characteristics to take part in the validation study. The calculation of the sample size was guided by the item-to-respondent ratio (1:30) (i.e., for each item questionnaire thirty respondents are needed) [31]. Adult patients (age ≥18 years), with a minimum of one chronic condition, treated with medications for at least 3 months, and with no cognitive and communicative disabilities were included in the study. Patients who could not communicate in Arabic or with cognitive impairments were excluded from this study.

Eligible participants were invited to participate and a verbal informed consent was obtained before participation. Participants were also asked to provide a contact number to be contacted 2 weeks later by phone call for the test-retest reliability. A data collection form was used to collect information from the participants.

### Data collection

The data collection tool was composed of four parts: 1) demographics characteristics, 2) clinical and medication histories, 3) the Morisky, Green and Levine (MGL) Medication Adherence Scale and 4) the Arabic Medications Adherence Assessment Tool (AAAT). The AAAT was used for convergent validity.

### Ethical procedure

Ethical approval was obtained from the Institutional Review Board of the site hospital (Ref. 2020/215). The IRB committee approved the verbal consent procedure as the research involved no interventions and presented no harm to the participants. In addition, the verbal consent procedure helped to avoid spread of COVID-19 virus from sharing papers and pens needed during the written consent procedure. The patients’ names, their phone numbers and the date of data collection were recorded on the data collection forms. All the data was appropriately coded and analyzed anonymously.

### The Morisky, Green and Levine (MGL) Medication Adherence Scale

The MGL scale is a 4-item generic, medication-adherence scale firstly developed in 1986 from an original 5-item tool [11]. Initially, it has been validated on 290 hypertensive patients showing an adequate reliability and internal consistency (Cronbach alpha = 0.61). It has been later used for the detection of nonadherence in other several health conditions in both Arabic-and non-Arabic speaking contexts [5–7, 13–29].

The scale encloses four questions: 1) “Do you ever forget to take your medicine?”, 2) “Are you careless at times about taking your medicine?”, 3) “When you feel better do you sometimes stop taking your medicine?” and 4) “Sometimes if you feel worse when you take the medicine, do you stop taking it?”. The theory underlying this measure is that medication nonadherence could occur due to forgetting, carelessness, stop taking the drug when the patient feels better or when the patient feels worse. The scale’s design facilitates the identification of problems and barriers to adequate adherence. It has a scoring scheme of “Yes” = 1 and “No” = 0 for each item with a total score ranging from 0 to 4. The sum of “Yes” answers provides a combined measure of nonadherence. Lower scores indicate higher level of adherence and the total patients’ scores can be categorized into high adherence level (0 item answered “yes”), moderate adherence level (1–2 items answered “yes”) and low level of adherence (3–4 items answered “yes”) [11].

### Scale translation

The English MGL tool was initially translated forward to Arabic and then backward into English. Each translation process was carried out by two independent bilingual individuals with a medical background. The final translated version (English) of the questionnaire was compared to the original one and the aforementioned forward-backward translation steps were carried out until no changes were further suggested to the back-translated questionnaire. The final Arabic version of the questionnaire was piloted on 30 respondents to check for clarity and easy understanding of the tool. Following the piloting phase, the questionnaire resulted to be clear and no changes were performed on it. Since the same methodology and sampling frame were followed for both the pilot and the main studies, the data collected from the 30 respondents was included into the final analysis [32].

### Data analysis

Data analysis was carried out using SPSS and AMOS (versions 26). All hypotheses testing were two-tailed with *p*-value <0.05 indicating statistical significance. The sociodemographic properties of the participants were presented as frequency and percentage for categorical variables and median and interquartile range (IQR) for continuous variables.

#### Reliability and internal consistency

Internal consistency of the MGL scale was evaluated using Cronbach’s alpha. Alpha values indicate the ability of the tool to assess the scale’s attributes intended to be measured. Values above 0.7 are usually used to indicate good measure; nevertheless, there is lack of agreement regarding the acceptable cutoffs [33].

#### Test-retest reliability

The test-retest reliability was used to measure the consistency of participants’ responses upon a repeated administration of the scale. The adherence questionnaires were used to collect responses a second time from 124 participants after a period of 2 weeks from the first interview. Test-retest reliability was evaluated via interclass correlation (ICC) and Spearman product moment correlation coefficient (Spearman r). Spearman r values ≥ 0.75 indicate very good to excellent correlation, values of 0.51–0.75 indicate moderate to good correlation, values of 0.26–0.50 indicate small correlation while values of 0–0.25 reflect little to no correlation [34].

#### Construct validity

Construct validity was conducted using exploratory factor analysis (EFA) followed by varimax rotation. This was performed to test the degree to which the MGL scale reflects the level of adherence to medication. As a result, Kaiser-Meyer-Olkin (KMO) test and Batlett’s test of sphericity were estimated. Parallel analysis and scree plots were conducted; factors with eigenvalues > 1 and factor loadings ≥ 0.4 were used.

#### Convergent validity

Convergent validity was estimated using Pearson’s correlation to correlate the Arabic translation of MGL scale to the preexisting validated tool AAAT (32) The AAAT is a 5-item Arabic assessment tool used to measure adherence to medications in the outpatient setting. It has been validated among Jordanians showing high internal consistency (Cronbach’s alpha = 0.80) [35].

#### Model fit

Confirmatory factor analysis (CFA) was used to evaluate the model fit. Goodness of fit indices included: Goodness of Fit Index (GFI) and Adjusted GFI (AGFI) where values ≥ 0.95 and ≥ 0.9, respectively are considered acceptable. It also included Normed Fit Index (NFI) with cut-off value for good fit ≥ 0.95, Root Mean Square Error of Approximation (RMSEA) and Standardized Root Mean Square Residual (SRMSR). RMSEA and SRMSR values < 0.06 and < 0.08 respectively are considered acceptable [36].

#### Linear regression

A multivariate linear regression was performed to investigate the presence of associations between the MGL scale scores and patients’ demographics and clinical characteristics. Factors showing significant correlations with the MGL scale scores (*p*<0.05) by the simple linear regression were included in the multivariate regression. Factors tested included age, number of medications, frequency of outpatient clinic visits and whether the patient has experience adverse effects (ADEs) from medication or not.

## Results

### Demographics

A total of 201 patients, including the pilot sample, were interviewed and their responses collected. Table 1 shows the demographic characteristics of the respondents. The median (IQR) age of all the participants was 57.0 [15], of them 58.2% were female. Most of the study participants (96%) were living with a family member or a caregiver; 78.6% were married. Almost half of the respondents (49.3%) received a diploma or higher degree of education (MCs or PhD) while only 20.9% were employed. Health care-related education and health care-related occupation were reported by 19.9% and 5% of all the participants respectively. Most of the participants (93.5%) resided in an urban area and had a monthly income of less than 600 USD (67.2%); a third (33.3%) reported having 3 family members or less.

**Table 1:** Demographic characteristics of the study population (N = 201).

Variable^a^	Frequency (%)	*p-value* ^c^
**Age** ^**b**^	57.0 (15)	**0.008**
**Gender**		0.765
Female	117 (58.2)	
Male	84 (41.8)	
**Marital Status**		0.324
Married	158 (78.6)	
Others	43 (21.4)	
**Living Conditions**		0.832
With a family member/Caregiver	193 (96)	
Alone	8 (4.0)	
**Education**		0.603
Diploma or higher degree (MSc or PhD)	99 (49.3)	
Scholar Degree	102 (50.7)	
**Health are Related Education**		0.083
No	161 (80.1)	
Yes	40 (19.9)	
**Occupation**		0.359
Employed	42 (20.9)	
Unemployed/Retired	159 (79.1)	
**Health Care Related Occupation**		0.598
No	191 (95)	
Yes	10 (5)	
**Location of Residence**		0.877
Urban	188 (93.5)	
Rural	13 (6.5)	
**Monthly Income**		0.949
≤ 600 USD	135 (67.2)	
> 600 USD	66 (32.8)	
**Number of Family Members**		0.130
≤ 3	67 (33.3)	
˃ 3	134 (66.7)	

^a^ All data is expressed as frequency (%) of participants unless otherwise indicated

^b^ Data described as median (IQR)

^c^ Simple linear regression of the variable’s association with MLG scale total score

Bold values indicate statistical significance *p*<0.05

### Clinical characteristics and medication adherence of the study population

**Fig 1:**
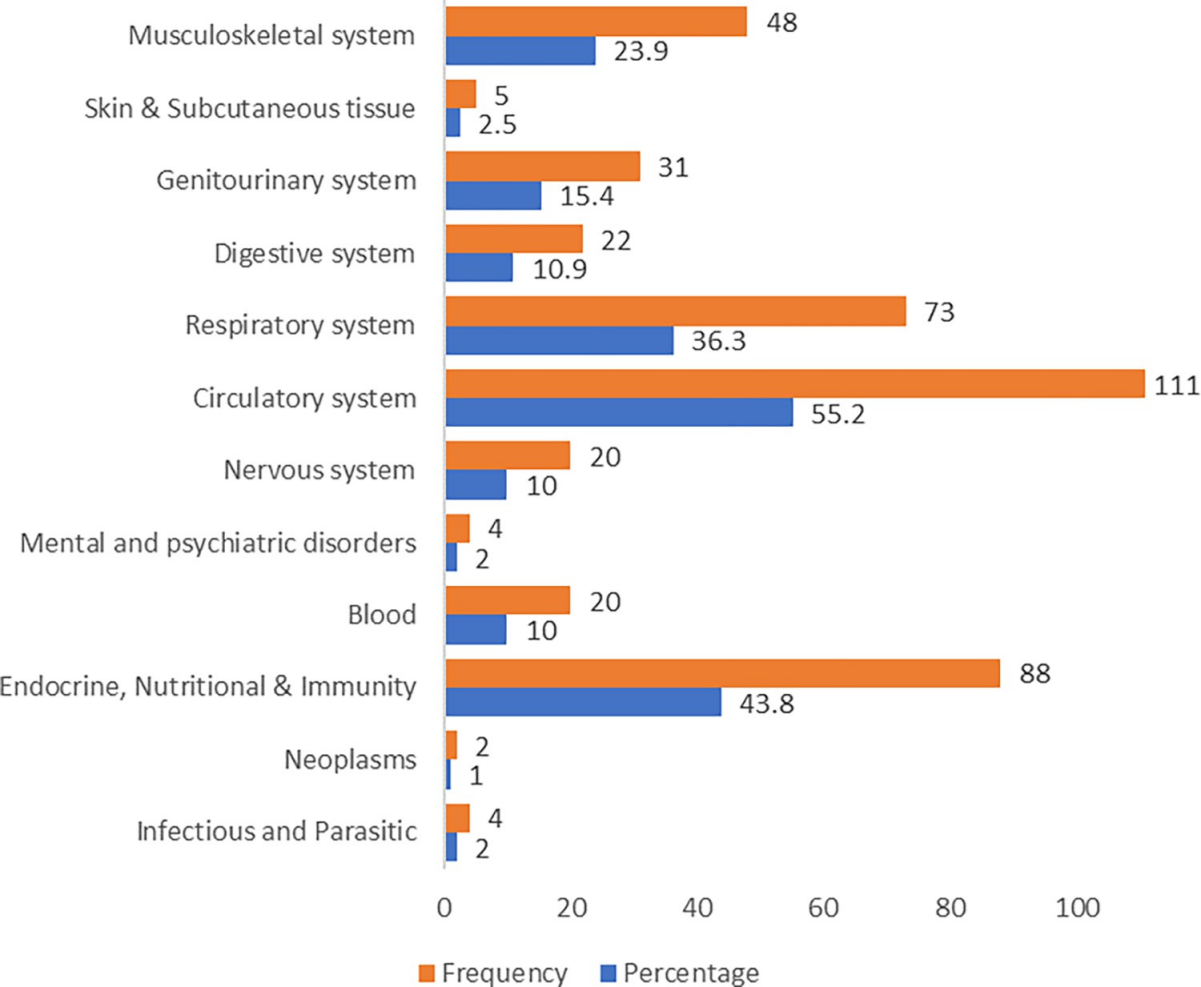
Frequency and percentages of chronic diseases among the study population (N = 201).

Almost half of the study population (59.2%) reported having two or less chronic conditions and taking 4 or less chronic medications concomitantly (56.2%). Circulatory, endocrine and respiratory diseases were the three most common chronic conditions affecting the study population (Fig 1). When asked about the frequency of regular outpatient clinic visits, most of the participants (93.0%) were appointing the clinic every 6 months or more frequently. Less than third of the study population (23.9%) reported having experienced ADEs from their medications.

Of all the participants, 20.9% had high medication adherence scores, almost half (59.2%) had moderate level of adherence, while low level of adherence was observed in 19.9% of the population. The patients’ AAAT scores were categorized into higher scores (˃10) indicating poor adherence to medication and lower scores (≤10) indicating good adherence. When applying this scoring criteria, 71.1% of the patients had good adherence to their medication, while 28.9% were low adherent. The clinical characteristics and the medication adherence scores of the study population are showed in Table 2.

**Table 2:** Clinical characteristics and medication adherence of the study population (N = 201).

Variable^a^	Frequency (%)	*p-value* ^c^
**Number of chronic diseases**		0.520
≤ 2	119 (59.2)	
˃ 2	82 (40.8)	
**Number of Medications**		**0.006**
≤ 4	113 (56.2)	
> 4	88 (43.8)	
**Frequency of outpatient clinic visits**		**0.001**
Less than every 6 months	14 (7.0)	
Every 6 months or more	187 (93.0)	
**Had experienced ADEs due to medications**		**0.001**
Yes	48 (23.9)	
No	153 (76.1)	
**MGL scale**		
High adherence level (0 item answered Yes)	42 (20.9)	
Moderate adherence level (1 or 2 item/s answered Yes)	119 (59.2)	
Low adherence level (3 or 4 items answered Yes)	40 (19.9)	
**AAAT**		
Good adherence (score ≤10)	143 (71.1)	
Poor adherence (score ˃10)	58 (28.9)	

^a^ All data is expressed as frequency (%) of participants unless otherwise indicated

^c^ Simple linear regression of the variable’s association with MGL scale total score

Bold values indicate statistical significance *p*<0.05

**Fig 2:**
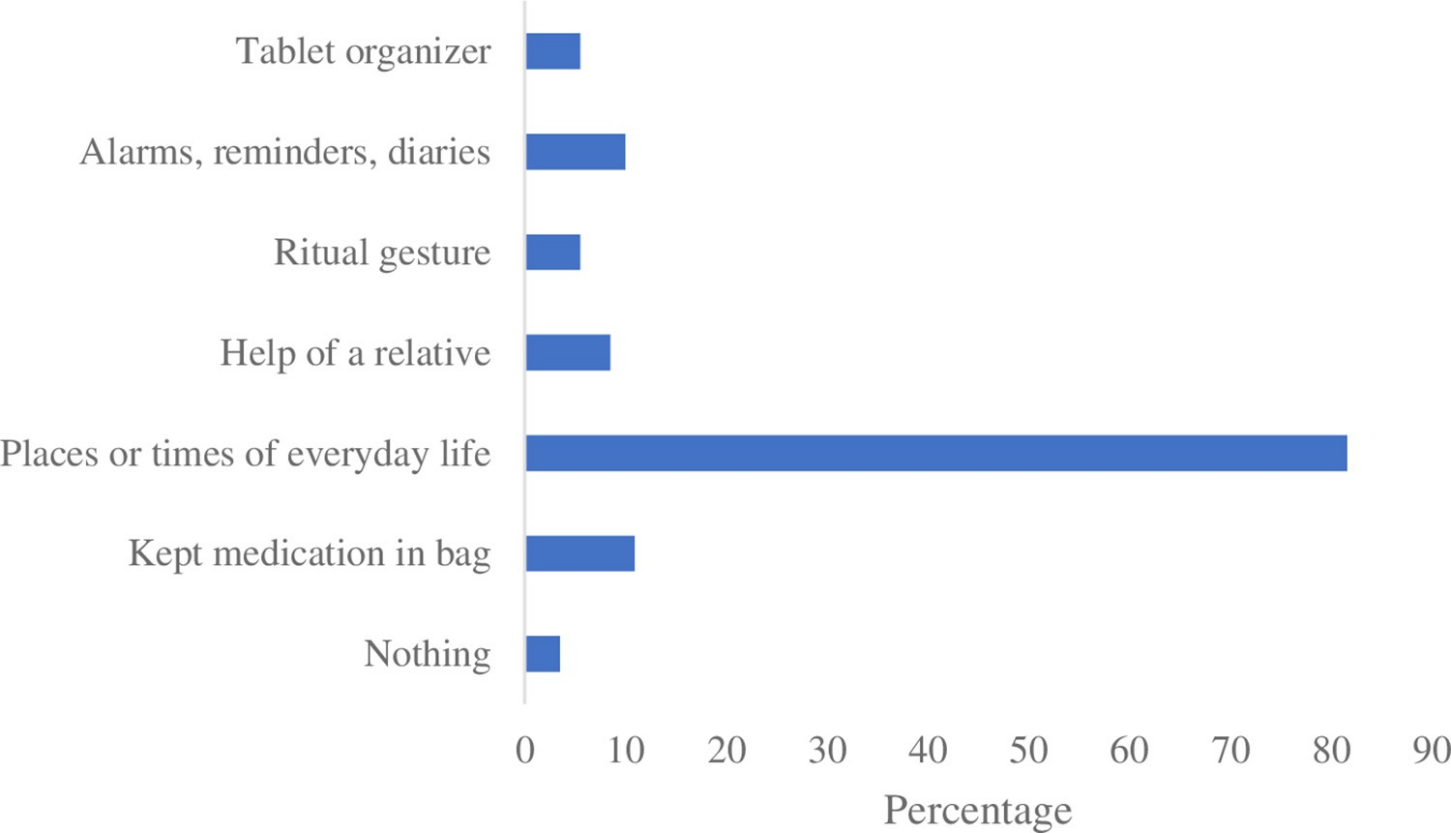
Strategies adopted by the participants to remember taking their medications.

Respondents were also asked about the strategies usually adopted to remind them taking the medication on time. The most (81.6%) were relying on times of everyday life, like woke up, meals, prayer, bed time to remember taking their medications (Fig 2).

### Reliability

Cronbach’s alpha and standardized Cronbach’s alpha of the Arabic version of the 4-item MGL scale were 0.593 and 0.613, respectively. As shown in Table 3, Cronbach’s alpha if specific item is deleted, ranged between 0.243–0.500, while the item correlation to total score range was 0.146–0.406.

**Table 3:** Reliability (N = 201).

Item Number	Corrected item-total correlation	Cronbach’s alpha if item deleted
1	0.195	0.448
2	0.406	0.243
3	0.322	0.327
4	0.146	0.500

Cronbach’s alpha for the 4 items = 0.593, Cronbach’s alpha based on the standardized items = 0.613

Table 4 shows the test-retest reliability measures for 124 participants. A significant strong ICC (x = 0.593, *p*<*0*.01) was detected and a significant excellent Pearson’s correlation (r = 0.889, *p<0*.01) was generated as well between responses at time 1 and time 2.

**Table 4:** Test-retest assessment using ICC and Pearson correlation between time 1 and time 2 (N = 124).

Scale	Intraclass correlation coefficient (95%CI)	Spearman correlation coefficient (r)
MGL scale	0.593 (0.458–0.707)*	0.889*
AAAT	0.765 (0.710–0.813)*	0.839*

*Correlation is significant at the 0.01 level (2-tailed).

The results also demonstrated a strong significant ICC and Pearson’s correlation between the test-retest responses for the AAAT.

### Construct validity

**Fig 3:**
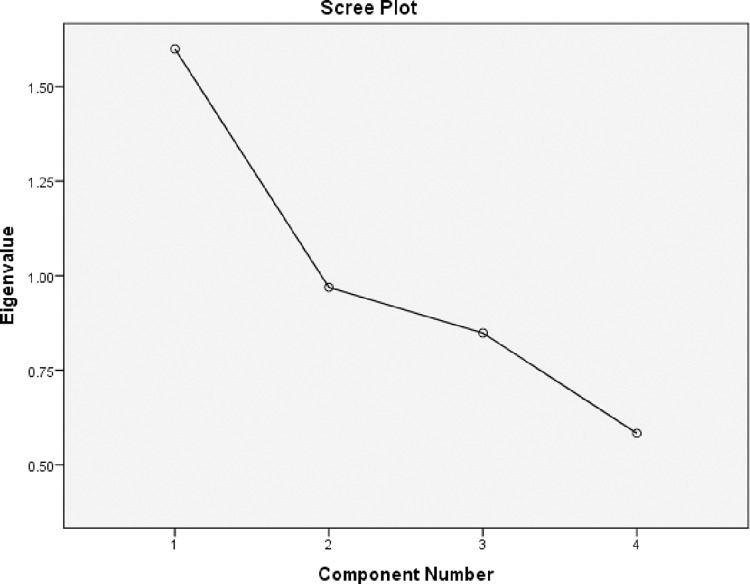
Scree plot the component number against eigenvalue (N = 201).

EFA revealed a unidimensional scale as only one component has eigenvalue > 1, this was also assured by the scree plot as showed in Fig 3. This component explained almost 40% of the total variance. Factor loadings of minimum value around 0.4 were recoded (Table 5). KMO value (0.583) proved sample adequacy and significant Bartlett’s test of sphericity (*p<0*.01) confirmed that variables are correlated. Consequently, data is suitable for CFA (Table 6).

**Table 5:** Patients answers to items and maximum factors loading, N = 201.

Item Number	Patients answered “NO”* to item, Frequency (%)	Factor loading
1	124 (61.7)	0.516
2	45 (45)	0.794
3	42 (20.9)	0.738
4	89 (44.3)	0.397

*”NO” answers reflect adherence

**Table 6:** Assessment of the data collection tool validity and reliability, N = 201.

	MGL scale	AAAT*
KMO	0.583	0.742
Bartlett’s test of sphericity	52.600	314.582
Df	6	10
*p-value*	0.000	0.000

*Cronbach’s alpha for the AAAT = 0.765, Cronbach’s alpha based on the standardized items = 0.785

### Convergent validity

When comparing the 4-items MGL scale with the AAAT, the Pearson’s coefficient (r) revealed a significant strong correlation between the two adherence tools r = 0.742 (p-value <0.01).

### Model fit

CFA results corroborated goodness of fit to the suggested scale. Values of estimated indices are: GFI = 0.997, AGFI = 0.987, NFI = 0.981, RMSEA = 0.010 and SRMSR = 0.0182.

### Regression

The multivariate linear regression showed significant associations between the MLGS scores and the number of medications administered by the patients (*p* = 0.038), the frequency of patients’ visits to the outpatient clinic (*p* = 0.007) and the experience of adverse effects (*p* = 0.001) (Table 7). Better adherence was associated with higher number of medications, higher frequency of outpatient clinic visits and not having experienced ADEs.

**Table 7:** Multivariate linear regression results for factors affecting the scores of MGL scale.

	Mean Square	F	*p-value*
Model	8.48 (R = 0.37)	7.78	0.000
Variable*	**Standardized Beta**	**t**	***p-value***
**Age**	-0.04	-0.50	0.618
**Medications** (˃4 medication/≤4 medication)	-0.15	-2.09	0.038
**Frequency of outpatient clinic visits** (<2 times yearly/≥2 times yearly)	0.19	2.73	0.007
**Had experienced ADEs due to medications** (Yes/No)	0.23	3.48	0.001

## Discussion

This study translated and validated the Morisky, Green and Levine 4-item adherence scale into Arabic language to be cross-culturally adapted to the Arabic-speaking communities. The Arabic version of the MGL scale was found to be reliable and valid with similar psychometric properties to the original English version. For instance, Cronbach alpha was found to be 0.59 which is comparable to the original α value = 0.61 [11]. Although values above 0.70 usually indicate good internal consistency, lower values are also considered satisfactory (0.58–0.97) [37]. Exploratory factor analysis and scree plot revealed a unidimensional scale, suggesting the lower values of Cronbach alpha, observed for both the original and translated versions, to be a result of the limited number of questions rather than the scale being multidimensional [31].

The Arabic language adopted in this study is the identical official speaking-language for multiple countries in the MENA region, which allows the Arabic version of MGL scale to be used by physicians and researchers throughout Arabic-speaking healthcare contexts. It might be however appropriate to estimate the Cronbach alpha reliability each time the questionnaire is implicated in a research project.

The strong test-retest reliability measures indicate stability and consistency of the tool upon its repeated administration which further corroborate the reliability of the scale. Convergent validity also showed a significant strong correlation between the MGL scale and the AAAT tool. The measures for internal consistency, test-retest reliability, and construct validity all demonstrated reliability and validity of the AAAT with values being even higher than those observed with the MGL scale. This suggests the appropriateness of the AAAT to be used for convergent validity and comparison in the study.

The levels of medication adherence reported among the study population using the MGL scale are consistent with those reported using the AAAT. Consistency in the results exists also with data published in recent studies similarly investigating medication adherence among Jordanians using the same MGL scale and showing that most of the patients were non-adherent to their medication [5–7, 17]. The rate percentages showed in this study are comparable with those from other reports depending on whether the patients with moderate levels of adherence are considered adherent to their medication and thus combined to the high-adherence group (similar percentages with AAAT), or deemed non-adherent and summed to the low-adherence group (similar to published data). The number of patients highly adherent to their medication remains however low.

The multivariate regression showed that higher number of medications, more frequent outpatient clinic visits and the patient not experiencing ADEs from the medications were significantly associated with higher adherence scores.

Despite some data demonstrated and inverse association between the number of medications and the level of adherence others showed that medication adherence was higher with more prescriptions [38–41]. Although the complexity of treatment might be a reason for nonadherence, minimizing the total number of daily doses was found to be more important than reducing the total number of medications in encouraging adherence [3, 42].

In a recent nationwide population-based study the total number of administered medications showed an inverted U shape association with the level of adherence. A peak in adherence was observed with 3–4 medications up to a total of nine, after which it declined prominently [40].

Visiting the outpatient clinic as per scheduled is considered, per se, a form of adherence to healthcare recommendations. In addition, patients need to visit their outpatient clinic to obtain their prescription, thus, it was expected for patients visiting the clinics more frequently to be more adherent to their treatment regimen. On these bases, ‘visit adherence’ was showed to improve adherence to medication and it was used as a measure for adherence [43]. It is worth mentioning however, that visiting the clinics doesn’t mean that patients really take their medications, which underlines the importance to assess adherence regularly during each medical visit [40, 43].

Despite the optimal use of medications as per recommendations can reduce the risk of adverse events, ADEs represents one of the most important barriers to medication adherence [44, 45]. Perception and experience of ADEs can led to poor adherence, probably indicating patients’ effort to minimize drug-related problems [44]. In this regard, patients frequently report not to be informed by their physician about the potential medication ADEs, which highlights the importance of engaging in patients’ education as knowledgeable patients were demonstrated to be more adherent to their medications [4, 46].

Recruitment of the study population occurred through conventional sampling, which allowed the inclusion of patients with different chronic diseases; this might implicate the use of the Arabic version of the scale among different chronic conditions. Limitations of the study include possible overestimation of adherence rate due to desirability bias and relying on phone calls upon collection of information at time 2 for the test-retest reliability.

In conclusion, this validation study demonstrates the Arabic version of the 4-item MGL adherence scale to be a valid and reliable tool to assess medication adherence among Arabic-speaking communities. The scale is a unidimensional tool, identifying the main barriers to nonadherence, with psychometric properties comparable to the original version. It is easily accessible to researchers and healthcare providers. In addition, this scale is the shortest and easiest to use among the others making it a great tool to be easily integrated within the medical visit to detect nonadherence in busy clinical settings. Implementing interventions targeting the patients with less compliance to regular clinic visits and those at higher risk of experiencing medication side effects, can have the greatest impact in optimizing medication adherence.

## Supporting information

S1 Table Arabic version of the MGL scale. DOI: 10.6084/m9.figshare.20228133.
(DOCX)
