# Supplementary material for: Translation and validation of the Arabic version of the Morisky, Green and Levine (MGL) adherence scale
Source: PLoS One. 2022 Oct 7;17(10):e0275778. doi: 10.1371/journal.pone.0275778 (PMC9543961; doi:10.1371/journal.pone.0275778)
Supplement: S1 Table — DOI: 10.6084/m9.figshare.20228133. (DOCX) [file pone.0275778.s001.docx]

| **Arabic Translation of MGL scale** | **4-item MGL scale (Morisky et al., 1986)** |  |
| --- | --- | --- |
| هل سبق و نسيت تناول دوائك؟ | Do you ever forget to take your medicine? | Q1 |
| هل احيانا لا تهتم بتناول دوائك؟ | Are you careless at times about taking your medicine? | Q2 |
| عندما تشعر بتحسن ،هل أحيانًا تتوقف عن تناول دوائك؟ | When you feel better do you sometimes stop taking your medicine? | Q3 |
| أحيانًا إذا شعرت بسوء عند تناول الدواء, هل تتوقف عن تناوله؟ | Sometimes if you feel worse when you take the medicine, do you stop taking it? | Q4 |

**Supporting information**

**S1 Table. Arabic version of the MGL scale**
